# Study on the Friction and Wear Properties of Multiple Rare-Earth-Oxide-Reinforced Resin-Based Friction Materials

**DOI:** 10.3390/ma17122990

**Published:** 2024-06-18

**Authors:** Lirong Huang, Minjie Huang, Junhua Du, Zhigang Liu, Wei Li, Jiangbo Zhu

**Affiliations:** 1 School of Mechanical and Electrial Engineering, Jiangxi University of Science and Technology, Ganzhou 341000, China; hmj081900@163.com (M.H.); 18279026603@163.com (J.Z.); 2 Jiangxi Huawu Brake Co., Ltd., Fengcheng 331100, China; lzg@hua-wu.com (Z.L.); liwei@hua-wu.com (W.L.)

**Keywords:** friction material, rare earth oxides, friction and wear, mechanical properties

## Abstract

Aiming to solve the problem of thermal decay of resin-based friction materials at high temperatures, rare-earth-lanthanum-oxide-/cerium-oxide-reinforced resin-based friction plates were prepared using a hot-pressing molding process. The effect of lanthanum/cerium oxide with different contents on the mechanical and tribological properties of the resin-based friction of materials was studied, and its mechanism was explored. The result shows that lanthanum/cerium oxide improves the mechanical and tribological properties of materials so that the coefficient of friction of the specimen is more stable on adding lanthanum/cerium oxide at 5% and 1%. Lanthanum/cerium oxide improves antidegradation properties of resin-based material and reduces the high-temperature wear rate by enhancing the interfacial effect so that the wear form of the specimen changes from predominantly adhesive wear to predominantly abrasive wear.

## 1. Introduction

As a clean, green, and renewable energy source, wind energy can help reduce the damage that coal shortages and power generation processes do to the environment [1,2]. The friction sheet in wind power generators needs to have a long service life and a stable friction coefficient. The features of resin-based friction materials ensure a simple process, low cost, and strong structure. The material is now the most in-demand class of composite material [3,4]. A friction material is usually a composite material made up of a combination of more than 10 components. These components are divided into four main parts: the binder, reinforcing fibers, friction modifiers, and fillers [5,6,7,8]. Nevertheless, the tribological properties of the material are significantly reduced because of severe “thermal degradation” of the resin at high temperatures [9,10,11]. Thus, it is of practical significance to improve the high-temperature degradation and tribological properties of resin-based friction materials.

Rare earth elements, because of their unique 4f electron layer structure, which provides a huge advantage in the field of high-tech materials, are renowned as a treasure house of new materials. These elements are called “industrial vitamins” and the “monosodium glutamate” of modern industry [12,13,14,15]. Studies show that the addition of rare earth elements to materials can be effective in improving their tribological properties. Jiang Wei et al. [16] studied the effect of lanthanum oxide on the performance of resin-based friction materials. The result shows that lanthanum oxide can improve the friction coefficient of the material. When the content of lanthanum oxide is 21.6%, the comprehensive performance of the material reaches its best. He Fushan et al. [17] prepared composites using lanthanum oxide and found that the friction coefficient of the modified material increased by 20.6%, and the wear rate was reduced by 43.8%. Zheng Kaikui et al. [18] used lanthanum chloride to prepare a rare earth solution and treat bamboo fibers. The results showed that the heat resistance of the modified bamboo fibers substantially increased, which further improved the high-temperature tribological properties of the material. Sun Jiachen et al. [19] modified resin-based friction materials using rare earth composite nanomaterials and found that, when cerium oxide and yttrium oxide were 1% and silica nanoparticles were 4%, the friction coefficient of the material improved, and the amount of wear reduced. In a study by Manroo et al. [20], metal matrix composites containing alumina nanoparticles and cerium oxide nanoparticle surfaces were fabricated by friction stir processing, respectively, and the results showed that the cerium-oxide-containing composites had the best wear-resistant properties as compared to the other fabricated composites.

When scholars study the influence of rare earth oxides on the properties of resin-based friction materials, the rare earth oxides used are relatively single, and the reinforcement material in the material formula is only a certain kind of fiber. In this paper, the authors used fibers combined with high-strength whiskers as the reinforcing components and used rare earth lanthanum oxide/cerium oxide for improving the performance of resin-based composites. The aim of creating these combinations was to investigate the effect of rare earth lanthanum oxide/cerium oxide on resin-based friction disks, explore the modification mechanism, and provide an experimental basis and theoretical guidance for preparing high-performance friction disks.

## 2. Experimental

### 2.1. Material Preparation

In this study, aramid fiber (para-aramid fiber “Twaron”, type 1099), ceramic fiber, potassium hexatitanate whisker, and calcium sulfate whisker were used as the main reinforcing materials. The aramid fiber of length 1.13 mm was provided by Teijin Group (Osaka, Japan). Ceramic fiber was provided by Jiangsu Ruike High-Tech Material Co., Ltd. (Huaian, China), with a diameter of 1–15 μm. Potassium hexatitanate whisker was provided by Shanghai Kaishefeng Industry Co., Ltd. (Shanghai, China), with a diameter of 0.4 μm. Calcium sulfate whisker was provided by Zhengzhou Bocelli Ecological Engineering Co., Ltd. (Zhengzhou, China), with a length-to-diameter ratio of 40–60 μm. A phenolic resin was used as the binder (the phenolic resin type PR-50099 has a flowability of 20 mm/125 °C, a cure time of 110 s/150°, and a hexamine content of 9%), and the modified materials were lanthanum and cerium oxides. The phenolic resin was provided by Nantong Sumitomo Bakelite Co., Ltd. (Nantong, China), with a mesh of 200–250. Lanthanum and cerium oxides were provided by Ganzhou Liantuo New Materials Co., Ltd. (Ganzhou, China), with a diameter of 10–20 μm. The formula of the friction material is shown in Table 1.

The other ingredients were zirconium silicate—15 wt%, barium sulfate—12 wt%, calcium hydroxide—2 wt%, flake graphite—8 wt%, and diatomaceous earth—10 wt%. Zirconium silicate was provided by Chongqing Jinjuyuan New Material Technology Co., Ltd. (Chongqing, China), with a mesh of 400. Barium sulfate was provided by Wuxi Mesd Chemical Products Co., Ltd. (Wuxi, China), with a mesh of 1250. Calcium hydroxide was provided by Tianxuan Composites (Shanghai) Co., Ltd. (Shanghai, China), with a mesh of 170. Scaled graphite was provided by Shenzhen Hanhui Graphite Co., Ltd. (Shenzhen, China), with a mesh of 100. Diatomaceous earth was provided by Guangzhou Shengzhipeng Trading Co., Ltd. (Guangzhou, China). Diatomaceous earth was mainly used as filler, in which silica ≥ 87 wt%, iron oxide ≤ 0.005 wt%, and ablation was 0.02 wt%.

### 2.2. Specimen Preparation

The specimens were prepared using the hot compression molding technique in the following steps:

(1) An electronic scale was used to weigh each raw material according to the formula in Table 1;
(2) First, aramid fiber was added into a JHF-20 mixer (Zhengzhou jinhe Powder Technology Co., Zhengzhou, China) for 5 min, and then the aramid fiber was opened and dispersed. Then, the other raw materials were put into the mixer for 15 min, and the rotating speed of the blades was 1000 r/min;
(3) The raw materials were mixed and put into the JFY60 hot press (Jilin jida Electromechanical Equipment Co., Changchun, China). The working pressure was set at 15 MPa, and the temperatures of the upper, middle, and lower molds were 150 °C, 160 °C, and 150 °C, respectively. The hardening time was 600 s. Finally, the pressed samples were put into a drying box for heat treatment at 180 °C for 12 h and then cooled down to room temperature. The heat-treated samples were cut using an MQD3220 cutting machine (Jiangsu Jinding Power Tools Group Co., Changzhou, China).

### 2.3. Testing Proceduce

**Figure 1:**
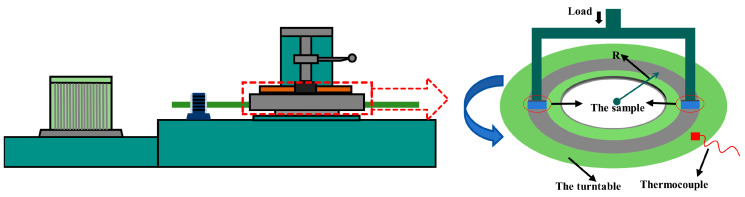
Schematic diagram of a friction wear tester.

Shear and pressure resistance tests were performed using the WAW600KN hydraulic universal testing machine (Guilin Ruite testing machine Co., Ltd., Guilin, China) in accordance with the China national standards GB/T 22309-2008 [21] and GB/T 10424-2002 [22]. The Rockwell hardness test was conducted using the XHR-15 plastic Rockwell hardness tester (Shanghai precision instrumentation Co., Ltd., Shanghai, China) in accordance with the China national standard GB/T 5766-2007 [23], where the diameter of the steel ball was 6.350 mm, the initial test force was 98.07 N, and the total test force was 588.4 N. The friction tests were performed using an XD-MSM constant speed tester (Xianyang Xinyi friction sealing equipment Co., Ltd., Xianyang, China) according to the China national standard GB 5763-2008 [24]. The testing machine schematic is presented in Figure 1. The specimen contact area had a size of 25 × 25 mm^2^ and a thickness of 5–7 mm. The counterpart disk radius was 0.15 m, the material was pearlitic gray cast iron HT250, the Brinell hardness was HB 180–220, and the disk speed was 490 r/min. The applied pressure on the specimens was retained at 0.98 MPa. Before the test, the disk was sanded with sandpaper with a grain size of P240 in JB/T 7498-2018 [25] so that its surface disk was free of obvious scratches, rust and pits, and other defects, and then cleaned with acetone. The friction coefficient and wear rate were calculated using a computer system with the following equations.

The friction coefficient was calculated at each test temperature according to Equation (1).

(1) $$\mu =\frac{f}{F}$$

In the formula, f represents the average friction; F represents the normal pressure.

The wear rate of each sample after 5000 revolutions at each temperature was calculated by applying Equation (2).

(2) $$V=\frac{1}{2\pi R}\times \frac{A}{n}\times \frac{d_{1}-d_{2}}{f_{m}}$$

In the formula, R represents the distance between the specimen center and the center of the disk rotation axis; n represents the total number of disk revolutions; A represents the total area of the friction surface of the specimen; $d_{1}$ represents the average thickness of the specimen before the test; $d_{2}$ represents the average thickness of the specimen after the test; and $f_{m}$ represents the total average friction.

Run in the cut sample on the testing machine until the degree of contact between the sample and the disk reaches 95% or more. Start by measuring the friction coefficient and wear rate of the sample from 100 °C to 350 °C and recording the values every 50 °C during the testing process. After the temperature rise test is completed, measure the friction coefficient and the wear rate of the sample within the range of 350 °C–100 °C for cooling. Using MLA650F field emission scanning electron microscopy (SEM) and energy-dispersive spectroscopy (EDS) (equipment produced by FEI Company in the United States), the wear morphology and surface elements of the samples were observed and analyzed. The MT-500 probe-type material surface abrasion tester produced by Lanzhou Zhongke Kaihua Technology Development Co., Ltd. (Lanzhou, China) analyzes the abrasion marks on the worn surface. The material surface was analyzed for the physical phase by an Empyrean-type X-ray diffractometer (XRD) manufactured by Panacor (Almelo, The Netherlands) in The Netherlands.

## 3. Results and Discussion

### 3.1. Mechanical Performance

**Figure 2:**
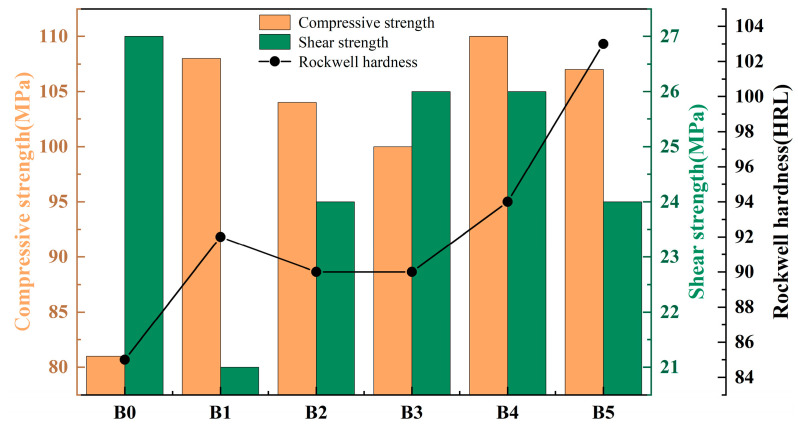
Mechanical properties of materials.

The Rockwell hardness, compressive strength, and shear strength of each group of samples were measured five times, and the average values were taken. The specific results are shown in Figure 2. From the result in Figure 2, it can be seen that rare earth lanthanum and cerium oxides increase the Rockwell hardness of resin-based friction materials. The hardness of the material is the maximum when the content of lanthanum oxide and cerium oxide is 5% and 1%, respectively, which is 21.17% higher than the hardness of the material without rare earth oxides. The overall compressive strength of the material improves in various ratio combinations of lanthanum oxide and cerium oxide. When the contents of lanthanum oxide and cerium oxide are 4% and 2%, respectively, the compressive strength of the material reaches a maximum value of 110 MPa, which is 35.8% higher than that of the samples without rare earth materials. However, rare earth elements have little effect on the shear resistance of the material. Among these, the shear strength of sample B1 is lower, at 21 MPa, whereas the shear strength of other samples remains stable, ranging from 24 to 26 MPa.

### 3.2. Tribological Performance

#### 3.2.1. Friction Coefficient

**Figure 3:**
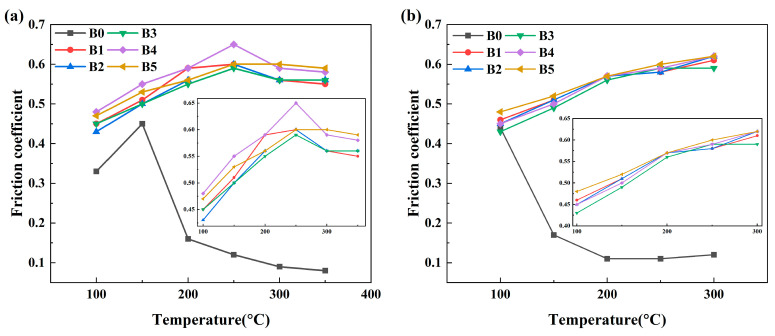
Friction coefficient variation of composite versus temperature: (**a**) fade process, (**b**) recovery process.

The effect of the ratio of rare earth lanthanum oxide and cerium oxide on the friction coefficient and stability of the brake material was tested at various braking temperatures, as shown in Figure 3. As shown in Figure 3a, the friction coefficient of the sample without lanthanum oxide and cerium oxide increases first and then decreases with increasing temperature. When the temperature exceeds 150 °C, the friction coefficient decreases significantly, and the material undergoes severe degradation. From B1 to B5, lanthanum/cerium oxide improves the overall friction coefficient of the material. The friction coefficient of the material increases first and then decreases with increasing temperature, and the friction coefficient reaches the maximum at 250 °C. On adding rare earth lanthanum and cerium oxides, the high-temperature friction coefficient of the specimens significantly improves. Although the friction coefficient of the materials slightly decreases in the range of 250 °C–350 °C, it is still higher than that of the B0 specimens, which indicates that the rare earth oxides significantly improve the antidegradation performance of the materials. This improvement occurs because rare earth elements have a unique electronic configuration and high chemical activity, and are prone to chemical reactions with many metal and nonmetallic elements. This is beneficial for the interaction between the components of the composite at the interface; the whole material system reaches the most stable state of thermodynamics, and the friction coefficient is improved at the macro level [26].

**Figure 4:**
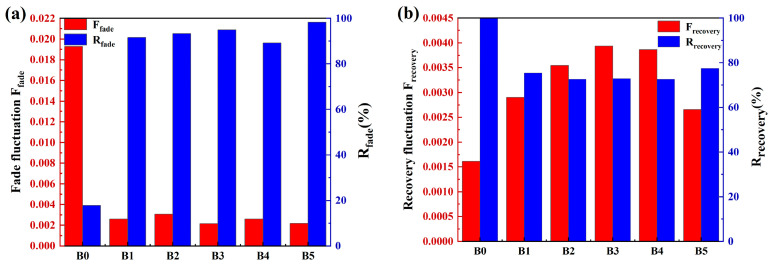
Materials’ friction coefficient, fade ratio, and recovery ratio variance: (**a**) fade process, (**b**) recovery process.

To better judge the stability of the material, the friction coefficient, fade ratio, and recovery ratio of each group of specimens were calculated. The results are shown in Figure 4. As shown in Figure 4a, the order of the friction coefficient stability during the fade process is B3 > B5 > B4 > B1 > B2 > B0. The friction coefficient variance is 0.00218 for the B5 specimen and does not appear to be highly different compared to the most stable B3 specimen. Additionally, the order of the fade ratio of the specimens is as follows: B5 > B3 > B2 > B1 > B4 > B0. Therefore, the friction stability of the B5 specimen is higher than that of the other five specimens in the process of material degradation. As presented in Figure 4b, the order of the friction coefficient stability during the recovery process is as follows: B5 > B1 > B2 > B3 > B4 > B0, of which B5 has the best stability. Furthermore, the recovery ratio is as follows: B0 > B5 > B1 > B3 > B4 > B2. The recovery rate of the B0 specimen is only for reference because of the serious failure of the B0 specimen. The friction coefficient variance and recovery ratio of B5 in the recovery are 0.00266 and 77.4%, respectively, with the best friction performance in the recovery stage. In summary, adding various ratios of rare earth lanthanum oxide/cerium oxide to the specimen can effectively improve the friction coefficient and stability of the materials. Among these, the best reinforcement effect is achieved when the ratio of lanthanum oxide to cerium oxide is 5:1.

#### 3.2.2. Wear Performance

**Figure 5:**
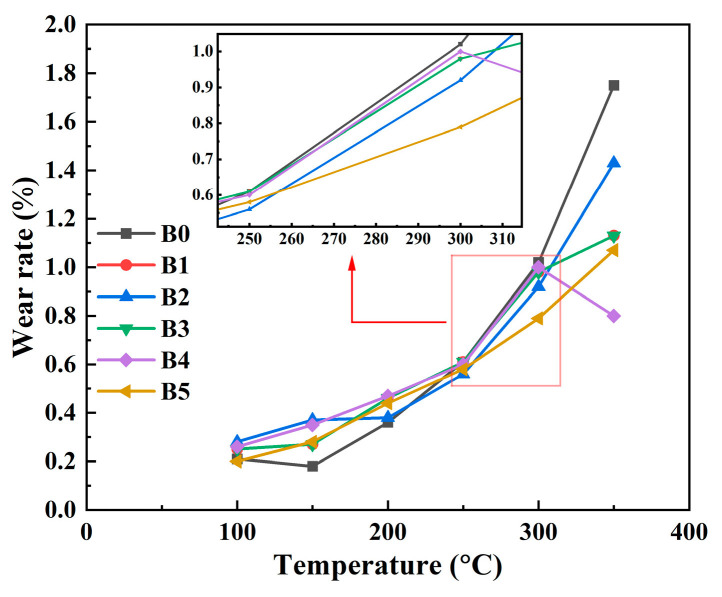
Variation of the wear rate with temperature.

The wear rate of each group of specimens was measured at various braking temperature, as shown in Figure 5. As presented in Figure 5, because of the increase in temperature on the sliding interface, the wear rate of all specimens increases with the increase in temperature. The wear rate of each group of specimens at the medium- and low-temperature (100–250 °C) stage is similar, whereas the B0 specimens show a sharp increase in the wear rate at the high-temperature (300–350 °C) stage. This is because decomposition of phenolic resin at high temperatures reduces the adhesion between fibers and whiskers as reinforcement components in the material and other materials, leading to intensified wear. The wear rate of the B1–B5 specimens is relatively low at the high-temperature (300–350 °C) stage.

**Figure 6:**
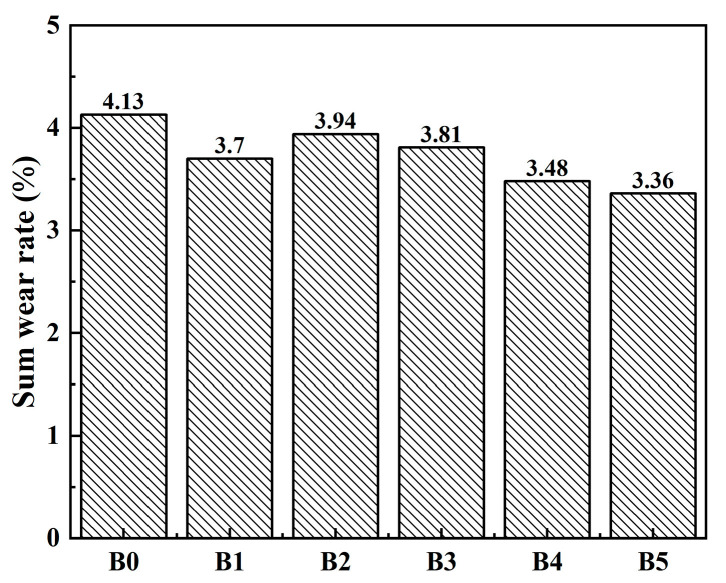
Wear rate sum of the samples B0–B5.

As shown in Figure 6, the total wear rate of the specimens without rare earth oxides is the highest, at 4.13%. The total wear rate of the B1–B5 specimens is lower than that of the B0 specimens. The total wear rate reaches the minimum when the contents of lanthanum and cerium oxides are 5% and 1%, respectively, which reduces the total wear rate by 18.6% relative to that of the B0 specimens. Because of the high activity of the phenolic hydroxyl group in the phenolic resin, it is easily oxidized and decomposed under medium- and high-temperature conditions, which leads to a decrease in the thermal stability of the resin [27,28]. The addition of rare earth elements reduces the wear rate of the material at high-temperature conditions. At the high-temperature stage, the rare earth elements can replace the hydrogen atoms in the phenolic hydroxyl group and coordinate the oxygen atom in the phenolic hydroxyl group. The generated coordination bond improves the resin strength. Simultaneously, the heat resistance of rare earth elements is high. The combination of these elements with phenolic resins slightly improves the thermal stability of the resins [29].

#### 3.2.3. Two-Dimensional Profile

**Figure 7:**
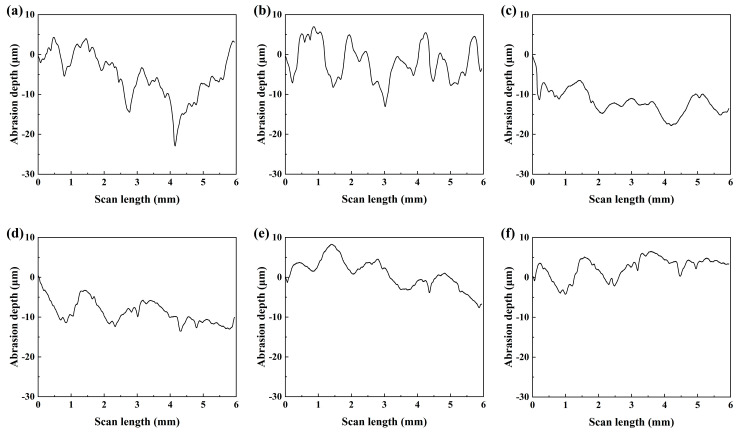
Two-dimensional topography of the wear surface: (**a**) B0; (**b**) B1; (**c**) B2; (**d**) B3; (**e**) B4; (**f**) B5.

To further investigate the effect of rare earth oxides on the frictional properties of brake materials at the microscopic level, microscopic scratch tests were carried out on specimens of rare earth oxides in various proportions, as shown in Figure 7. As shown in Figure 7, the maximum wear depth of the specimen without lanthanum oxide/cerium oxide is 22.9 μm. This indicates that the wear surface has deep grooves and is severely worn, and the wear mark depth is the largest compared with other specimens. As shown in Figure 7b–f, the depth of the abrasion marks of the specimens with rare earth oxides is shallower, and the depth of the abrasion marks of the specimen B5 is the smallest, followed by B4. The results of the microscopic test are the same as the wear of the specimens at the macroscopic level.

### 3.3. Worn Surface Analyses

**Figure 8:**
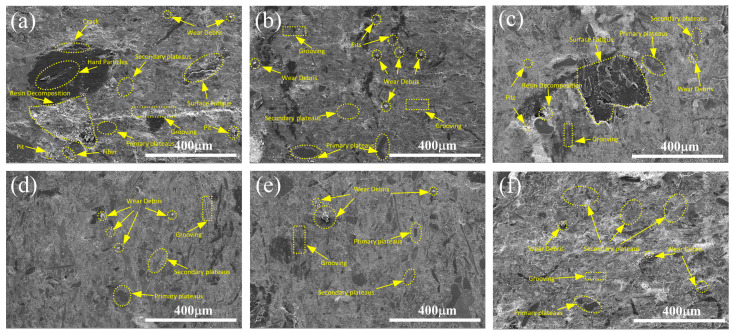
SEM micrographs of worn surface: (**a**) B0; (**b**) B1; (**c**) B2; (**d**) B3; (**e**) B4; (**f**) B5.

To investigate the effect and mechanism of various ratios of rare earth lanthanum oxide and cerium oxide on the surface wear morphology of brake materials, the surface morphology of the specimens after the friction wear test was observed, as shown in Figure 8. In Figure 8a, the specimen without rare earth oxides has obvious hard particles and part of the wear debris, causing an obvious scratch on the surface of the material and poor surface flatness. As the temperature increases, decomposition of the phenolic resin inhibits the bonding between fibers, whiskers, and the matrix, leading to an increase in the wear rate of the specimen. Some of the whiskers on the surface of the material are semi-exposed, with surface damage phenomena such as plowing grooves and surface fatigue. At this point, the form of wear is adhesive wear, with abrasive wear as a supplement. Thus, as the temperature of the material friction coefficient gradually decreases, the wear rate increases. As shown in Figure 8b–f, the surface of the specimens containing rare earth lanthanum oxide and cerium oxide does not show obvious phenomena such as plowing and scratching, and most of the specimens have good surface flatness. However, Figure 8c shows some flaking pits on the surface of the material, which results in relatively severe wear for this specimen. This observation is consistent with the wear measured in Figure 6.

Large areas of secondary platforms and a few primary platforms are formed on the surfaces of other specimen groups containing rare earth materials. The primary platform is composed mainly of components with high hardness in the material fixed on the sliding surface, which gradually wear because of pressure and friction. The latter is the friction layer formed by the abrasive debris generated near the primary platform under the action of pressure and temperature [30]. As shown in Figure 8f, a large continuous surface of the specimen with a small amount of abrasive debris indicates that the wear form of the specimen is dominated by abrasive wear and supplemented by adhesive wear. In combination with Figure 6, it shows that the total wear rate of the material is minimized under this wear mechanism. Many secondary platforms can avoid direct contact between the material and its counterpart during the wear process to improve the friction and wear properties of materials.

In conclusion, the addition of rare earth lanthanum/cerium oxide can improve the friction and wear performance of resin-based friction materials and increase the resistance to degradation. On the one hand, because of the large atomic radius of lanthanum and cerium elements (La: 0.187 nm, Ce: 0.182 nm, Si: 0.117 nm, C: 0.077 nm, Zr: 0.145 nm, O: 0.066 nm, Al: 0.118 nm), there is a strong positional competition with other elements in the material. It makes itself enriched at the grain boundary, which is suitable for large atoms to occupy. Additionally, this characteristic inhibits the growth of grains, makes the grains finer, and increases the area of the grain boundary, thus improving the interfacial bonding ability and promoting the formation of the friction film, reducing the abrasion rate of the material, and prolonging the service life. Combined with Figure 5, it can be seen that, in the high-temperature stage, the wear rate of the specimens with the addition of rare earth lanthanum/cerium oxide is lower overall than that of the specimens without the addition of rare earths, indicating that the oxide film formed by the addition of rare earth elements is relatively stable at high temperatures. On the other hand, the rare earth elements themselves have good heat resistance, and, when combined with the resin under high-temperature working conditions, they improve the thermal stability of the resin. This change in turn enhances the degree of adhesion between the resin and other components, and ultimately makes the material more resistant to degradation. From the wear morphology of the specimen surface, note that the wear degree of the specimen with added lanthanum oxide/cerium oxide is significantly less than that of the specimen without added rare earth oxide, and the effect of B5 specimen is the best.

### 3.4. Energy-Dispersive Spectroscopy Elemental and X-ray Diffractometer Phase Analysis

**Figure 9:**
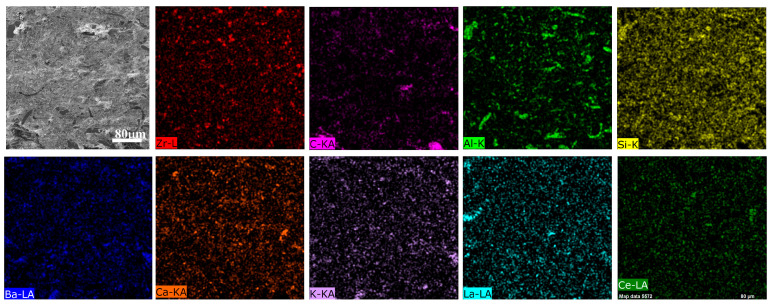
EDS of worn surface morphology of the B5 sample.

**Figure 10:**
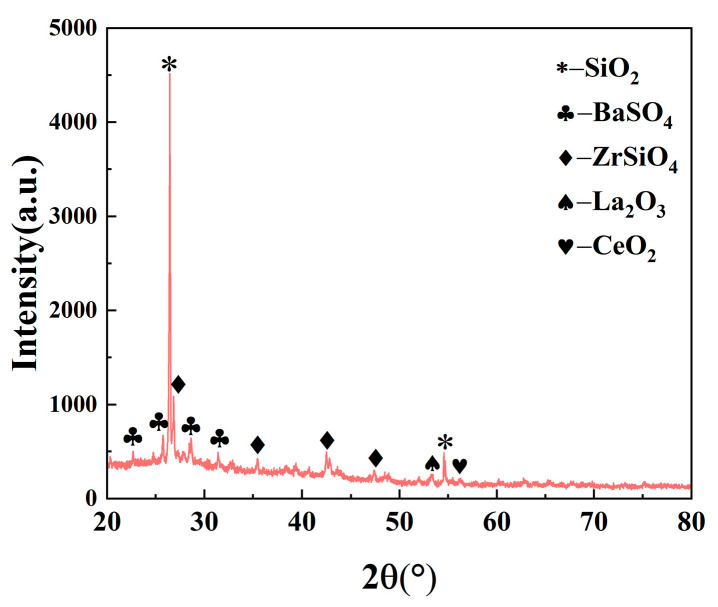
XRD pattern of friction surface of B5 sample.

To further understand the element distribution on the surface of the material as well as the composition of the physical phase, the surface of the B5 material was analyzed using EDS element scanning and XRD physical phase analysis. The results are shown in Figure 9 and Figure 10. As shown in Figure 9, the main elements of the primary platform on the surface of the material are Al and Si, indicating that the primary platform is mainly composed of Al_2_O_3_ and SiO_2_. The ceramic fiber and diatomite in the formulation contain a large amount of SiO_2_ such that the distribution of silicon elements that can be seen from the spectrum is quite dense, and the lanthanum and cerium elements are distributed relatively uniformly. However, because the ratio of lanthanum and cerium oxides in the formulation is 5:1, it makes the distribution density of lanthanum element relatively large. The uniform distribution of other elements such as barium and calcium indicates that the relevant materials are evenly distributed in the material, which is beneficial for improving the friction coefficient between friction partners.

The XRD diffraction pattern of the worn surface of B5 is shown in Figure 10, and the results indicate the presence of SiO_2_, BaSO_4_, La_2_O_3_, and CeO_2_ on the surface of the worn specimen. BaSO_4_, as a part of the friction performance regulator, has a stabilizing role in the friction coefficient of the material, whereas ZrSiO_4_, as a part of the friction-increasing filler, improves the friction coefficient of the material. Many high-hardness compounds, such as SiO_2_, are uniformly distributed in the material, increasing the Rockell hardness and compressive strength of the material. Lanthanum oxide and cerium oxide occupy a favorable position on the grain boundary by virtue of the large atomic radius of elements, which can inhibit the growth of grains, refine the grains, and improve the interfacial bonding ability. Moreover, lanthanum oxide and cerium oxide have high chemical activity, and the oxygen atoms in the phenolic hydroxyl group in the resin undergo a coordination reaction. The generated coordination bond improves the strength of the resin. Thus, lanthanum oxide and cerium oxide act as modifiers and, together, improve the friction and wear properties of materials.

## 4. Discussion

In this study, the friction properties of the sample were tested, and the worn samples were detected and analyzed by SEM, EDS, a two-dimensional profilometer, and an XRD. We found that rare earth materials can improve the overall friction coefficient of resin-based friction materials and reduce the wear rate at high temperature. As can be seen from Figure 8, the materials without added rare earth oxide showed obvious scratches and surface damage phenomena with poor overall flatness, and this wear mechanism was mainly based on adhesive wear. The wear surface of the added rare earth material did not show obvious furrows, and the flatness was relatively good, indicating that the rare earth material can promote the formation of the friction film on the surface material, and the wear mechanism is mainly based on abrasive wear.

In summary, rare earth materials can improve the antidegradation properties of resin-based friction materials and then enhance the tribological properties of the materials. This improves the field of reference for resin-based friction materials to a certain extent.

## 5. Conclusions

(1) The addition of rare earth lanthanum oxide and cerium oxide to the material can effectively improve the friction coefficient and reduce the wear rate, as well as improve the Rockwell hardness and compression resistance of the material. Among these, when the content of lanthanum oxide and cerium oxide is 5% and 1%, respectively, the specimens have the highest high-temperature friction coefficient, the wear is low and stable, and the total wear rate is reduced by 18.6%. Compared with that of the specimen without rare earth oxides, the surface wears uniformly, with the shallowest wear marks and optimal comprehensive tribological performance;
(2) The results show that the wear surface of the specimens without rare earth oxide shows flaking pits, obvious abrasion marks, and many abrasive debris, and the wear mode is mainly adhesive wear. However, the wear surface of most of the specimens with lanthanum/cerium oxides is relatively flat. When the content of lanthanum oxide and cerium oxide is 5% and 1%, respectively, many continuous secondary platforms and abrasive debris are formed on the surface of the specimen. The wear mode is dominated by abrasive wear and supplemented by adhesive wear. It indicates that lanthanum oxide/cerium oxide can increase the bonding strength of the resin and promote formation of a friction film on the surface of the material;
(3) At high temperatures, lanthanum oxide and cerium oxide can coordinate with the oxygen atoms in the phenolic hydroxyl groups in the resin. This coordination inhibits grain growth at grain boundaries and increases the area of grain boundaries. Therefore, the modified materials show better antidegradation performance and friction and wear properties under high-temperature conditions. This improves the service life of resin-based friction materials at high temperatures and also broadens their field of use.

## Figures and Tables

**Table 1:** Formula of friction materials.

Raw Materials (by wt%)	B0	B1	B2	B3	B4	B5
phenolic resin	17	11	11	11	11	11
aramid fiber	4	4	4	4	4	4
ceramic fiber	15	15	15	15	15	15
potassium titanate whiskers	12	12	12	12	12	12
calcium sulfate whisker	5	5	5	5	5	5
lanthanum oxide		1	2	3	4	5
cerium oxide		5	4	3	2	1
other	47	47	47	47	47	47

## Data Availability

The original contributions presented in the study are included in the article, further inquiries can be directed to the corresponding authors.
